# Associations between dietary fat-soluble vitamins and pulmonary function and airway inflammation: A cross-sectional study

**DOI:** 10.1097/MD.0000000000049160

**Published:** 2026-06-05

**Authors:** Ping Zhang, Shengsong Chen, Hanyan Xu, Yinshui Miao, Hui Yang, Qingyuan Zhan

**Affiliations:** a Department of Respiratory and Critical Care Medicine, The First Affiliated Hospital, Jiangxi Medical College, Nanchang University, Nanchang, Jiangxi, China; b Department of Respiratory and Critical Care Medicine, Shangrao People’s Hospital, Shangrao, Jiangxi, China; c National Regional Center for Respiratory Medicine, China-Japan Friendship Jiangxi Hospital, Nanchang, Jiangxi, China; d Department of Pulmonary and Critical Care Medicine, Center of Respiratory Medicine, China-Japan Friendship Hospital, No 2, East Yinghua Road, Chaoyang District, Beijing, China.

**Keywords:** airway inflammation, fat-soluble vitamins, lung health, NHANES, pulmonary function, vitamin A, vitamin D, vitamin E, vitamin K

## Abstract

Pulmonary function and airway inflammation are key indicators of respiratory health. Dietary factors, particularly fat-soluble vitamins, may be related to these outcomes, yet their associations in general populations remain inadequately characterized. We analyzed data from the National Health and Nutrition Examination Survey 2007 to 2012, including 9328 adults. Fat-soluble vitamin intake (A, D, E, K) was assessed via 24-hour dietary recalls. Spirometry measures (forced vital capacity, forced expiratory volume in the first second [FEV1], forced expiratory flow between 25% and 75% of vital capacity) and fractional exhaled nitric oxide (FeNO) were used to assess pulmonary function and airway inflammation, respectively. Multivariable linear regression and generalized additive models were employed to evaluate associations, with adjustment for sociodemographic, lifestyle, and clinical factors. In fully adjusted models, vitamins A and D were positively associated with forced vital capacity (*β* = 0.04 mL and 3.72 mL per µg/day, respectively). Vitamins A and K were positively associated with FeNO (*β* = 1.1 ppb and 3.3 ppb per unit increase, respectively). Exploratory analyses using generalized additive models identified nonlinear inverted U-shaped relationships for vitamins A and D with FEV1, with threshold points at 958 µg/day and 11.95 µg/day. Below these thresholds, each unit increase in vitamin A and vitamin D intake was associated with an increase in FEV1 of 0.07 mL and 4.15 mL, respectively. These threshold values are exploratory and require validation in future studies. Subgroup analyses indicated significant interactions with body mass index, particularly among overweight individuals. Dietary intake of vitamins A and D is positively associated with lung function, while vitamins A and K are positively associated with FeNO in a cross-sectional analysis. These findings highlight the complex associations between fat-soluble vitamins and respiratory health indicators, providing population-level observational evidence for future research on nutritional factors and lung health. Exploratory analyses suggest inverted U-shaped relationships between vitamins A and D and FEV1, but these findings are hypothesis-generating and require validation. No causal or preventive implications can be drawn from this cross-sectional analysis.

## 1. Introduction

Lung function and airway inflammation are critical factors in respiratory health. Markers such as fractional exhaled nitric oxide (FeNO) have emerged as valuable noninvasive biomarkers for assessing airway inflammation, particularly in conditions such as asthma, where they reflect eosinophilic inflammation and oxidative stress.^[1,2]^ Environmental and lifestyle factors can influence respiratory health, and dietary nutrients may act as modulators; however, the associations between fat-soluble vitamins and lung function or airway inflammation in the general population remain incompletely characterized. As the global incidence of lung diseases such as chronic obstructive pulmonary disease continues to rise, understanding the associations of dietary factors with respiratory health indicators is crucial for providing observational evidence for future research.^[1]^

Fat-soluble vitamins, particularly vitamins A, D, E, and K, are vital for maintaining various biological functions, including those pertinent to lung health. These vitamins exhibit antioxidant and anti-inflammatory properties, which are biological characteristics potentially relevant to airway and lung tissue homeostasis.^[3]^ For example, vitamin D influences surfactant metabolism and promotes epithelial-mesenchymal interactions, which are essential for lung maturation and function.^[4]^ Vitamin A has been linked to the regulation of extracellular matrix proteins involved in airway development and alveolarization.^[4]^ Despite growing interest, the quantitative associations between dietary intake of fat-soluble vitamins (A, D, E, K) and lung function or airway inflammation in the general adult population are not fully elucidated. Additionally, potential nonlinear relationships and subgroup differences (e.g., by body mass index (BMI) or smoking status) have not been systematically explored in large population-based studies, limiting the translation of nutritional evidence into respiratory health guidance.

In recent years, growing attention has been directed toward the relationship between diet and lung health, with several studies exploring how various nutrients may influence respiratory outcomes.^[5–7]^ These findings underscore the importance of nutritional factors in respiratory health research.

This observational cross-sectional study aimed to investigate the associations between dietary fat-soluble vitamin intake (A, D, E, K) and lung function (assessed via spirometry) as well as airway inflammation (assessed via FeNO) using data from the National Health and Nutrition Examination Survey (NHANES) 2007 to 2012. We further explored potential nonlinear relationships and subgroup differences to provide comprehensive observational evidence for nutritional research in respiratory health.

## 2. Materials and methods

### 2.1. Data source

This population-based cross-sectional study utilized data from NHANES, managed by the National Center for Health Statistics under the Centers for Disease Control and Prevention (http://www.cdc.gov/nchs/nhanes/). NHANES is designed to assess the health and nutritional status of adults and children in the United States (U.S.) using a complex, multistage sampling strategy to ensure representativeness of the noninstitutionalized U.S. population. The participants first complete a household interview and are then invited to a mobile examination center for comprehensive examinations, making the collected data reliable and multidimensional.^[8]^

### 2.2. Ethical approval and informed consent

This study was approved by the Ethics Committee of the First Affiliated Hospital of Jiangxi Medical College, Nanchang University (Approval No.: 202401041B1). The NHANES study was approved by the National Center for Health Statistics Research Ethics Review Board, and all participants provided written informed consent. Informed consent was waived for this secondary analysis of de-identified public data.

### 2.3. Study population

**Figure 1.**
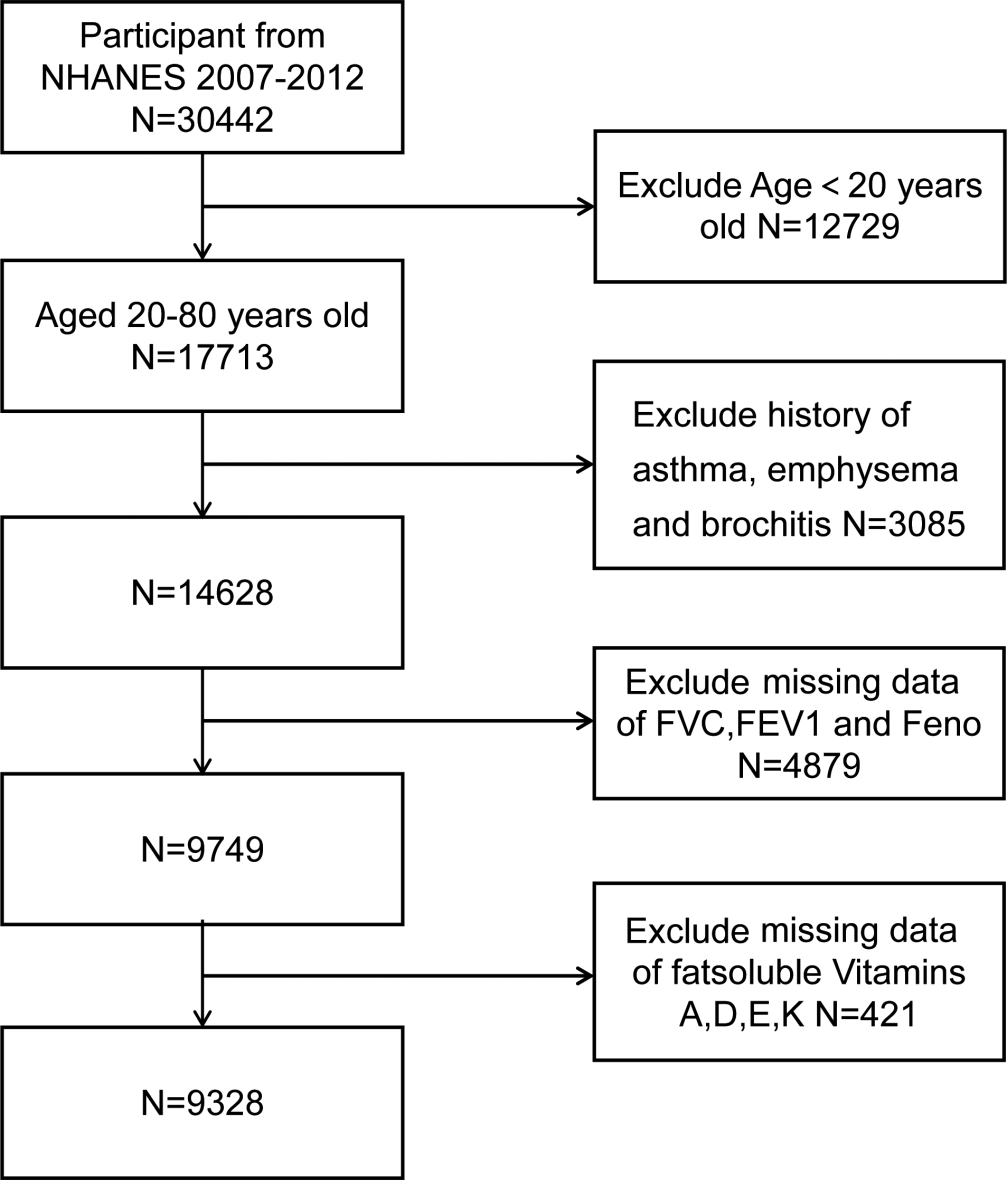
Flow chart of participant selection. FeNO = fractional exhaled nitric oxide, FEV1 = forced expiratory volume in the first second, FVC = forced vital capacity, N = number of participants, NHANES = National Health and Nutrition Examination Survey.

This study utilized survey data from the NHANES database for the years 2007 to 2012. A total of 30,442 participants were initially included. The exclusion criteria were as follows: participants under 20 years of age (N = 12,729); those with a prior physician diagnosis of asthma, emphysema, or chronic bronchitis (N = 3085); individuals with missing pulmonary function test data or FeNO measurements (N = 4879); and those with missing daily intake data for fat-soluble vitamins (N = 421). The final analysis included 9328 eligible adult participants (Fig. 1).

### 2.4. Study variables

#### 2.4.1. Assessment of daily intake of fat-soluble vitamins A, D, E, and K

Data on participants’ food intake over 2 nonconsecutive days were collected through 24-hour dietary recall interviews. The estimation of dietary nutrient intake was performed via the United States Department of Agriculture ’s Food and Nutrient Database for Dietary Studies. The first dietary recall interview was conducted at the mobile examination center, and the second interview was administered via telephone 3 to 10 days later. In this study, the daily intake of fat-soluble vitamins A, D, E, and K was calculated as the average of these 2 recordings. This approach may not fully reflect long-term usual intake of fat-soluble vitamins, which could introduce measurement bias. We calculated the average of the 2 recalls to minimize random error, but readers should interpret the findings in the context of short-term intake assessment.

#### 2.4.2. Pulmonary function and exhaled nitric oxide

##### 2.4.2.1. Spirometry

Spirometry was performed according to the data quality standards of the American Thoracic Society.^[9]^ The study indicators included forced vital capacity (FVC), forced expiratory volume in the first second (FEV1), and forced expiratory flow between 25% and 75% of vital capacity (FEF25–75%), all obtained from prebronchodilator spirometry. Only prebronchodilator spirometry data were analyzed because the study population excluded individuals with physician-diagnosed chronic respiratory diseases, and these values more accurately reflect baseline lung function in the general adult population, aligning with our research objective to assess inherent lung function.

##### 2.4.2.2. FeNO measurement

FeNO was measured via the Aerocrine NIOX MINO device (Aerocrine AB ), which employs an electrochemical sensor to detect nitric oxide levels in exhaled breath and provides integer values ranging from 5 to 300 ppb. According to the NHANES protocols, 2 reproducible FeNO measurements were required.^[9]^

#### 2.4.3. Covariates

We included clinically significant covariates, primarily sociodemographic characteristics, lifestyle and occupational factors, common underlying medical conditions, and total daily energy intake. The key sociodemographic characteristics included age (years), sex, race/ethnicity, family income–poverty ratio, marital status, educational level, and BMI (kg/m^2^). Lifestyle factors and underlying medical conditions included smoking (≥ 100 cigarettes in life), alcohol abuse (≥ 5 drinks every day), occupational exposure (ever exposed to dust or fumes), sedentary time, hypertension, and diabetes. Total daily energy intake (kcal/day) was estimated from the dietary interviews.

### 2.5. Statistical analysis

Analyses were conducted using R (version 4.5) and EmpowerStats (version 5.2), incorporating sampling weights per NHANES guidelines.^[8]^ A *P* value < .05 was considered statistically significant. Multicollinearity was assessed via the variance inflation factor (VIF), with a VIF < 5 as the inclusion criterion.

First, generalized linear models were employed to examine the associations between the 4 fat-soluble vitamins and pulmonary function parameters or FeNO. For sensitivity analysis, vitamins were modeled as quartiles, with the lowest intake quartile (Q1) as the reference. Three models were constructed: Model 1: unadjusted; Model 2: adjusted for age, sex, and race; Model 3: adjusted for all covariates listed above.

Generalized additive models (GAMs) with restricted cubic splines were used for exploratory analysis to investigate potential nonlinear relationships, as nutritional-health outcome relationships often exhibit dose-response patterns. If the linear association in Model 3 was not statistically significant but the quartile-based trend was, GAMs were applied to explore nonlinearity. Threshold effect analysis was performed using the maximum likelihood method with bootstrap resampling. These GAM and threshold analyses are exploratory, and their results should be interpreted cautiously.

Second, subgroup analyses were conducted using Model 3 to explore the consistency of associations and potential interactions across strata of age, sex, race, BMI, smoking status, and occupational exposure. Given the multiple comparisons involved, these analyses carry a risk of false-positive findings and are intended to be descriptive and hypothesis-generating.

## 3. Results

### 3.1. Baseline characteristics of the participants

Table 1 summarizes the characteristics of the 9328 participants. Higher FVC, FEV1, and FEF25–75% values were observed in younger individuals, males, Mexican Americans, those with higher education, non-excessive drinkers, individuals without diabetes or hypertension, and those with higher total energy intake. Lower values were observed in older individuals, females, other racial groups, those with BMI > 30, and those with occupational exposure. FeNO levels were higher in older adults, other racial groups, participants with higher education, those with BMI > 30, and nonsmokers.

**Table 1:** Weighted basic characteristics of the study population with FeNO and lung function parameters.

Characteristic		Total	FVC (mL)	FEV1 (mL)	FEF25–75% (mL/s)	FeNO (ppb)
		N = 9328 (100%)	Mean ± SD			
Age						
	20–39	3447 (36.9%)	4557.79 ± 1011.81	3743.17 ± 798.08	3908.22 ± 1114.87	15.73 ± 12.34
	40–59	3578 (38.3%)	4122.44 ± 1036.04	3184.48 ± 787.36	2926.98 ± 1073.75	16.70 ± 13.23
	≥ 60	2303 (24.6%)	3501.98 ± 988.24	2556.67 ± 705.64	1962.61 ± 895.39	18.87 ± 11.85
*P* value			< .001	< .001	< .001	< .001
Gender						
	Male	4870 (52.2%)	4866.27 ± 920.81	3792.69 ± 799.83	3508.89 ± 1345.20	18.25 ± 14.19
	Female	4458 (47.8%)	3441.06 ± 692.87	2744.51 ± 614.00	2726.98 ± 1055.14	15.08 ± 10.63
*P* value			< .001	< .001	< .001	< .001
Race						
	Mexican American	1633 (17.5%)	4145.80 ± 964.39	3368.17 ± 790.06	3562.77 ± 1188.06	16.62 ± 12.47
	Other Hispanic	1027 (11.0%)	4028.68 ± 1005.87	3277.16 ± 825.99	3439.92 ± 1162.24	17.52 ± 13.69
	Non-Hispanic White	3890 (41.7%)	4319.78 ± 1097.39	3358.00 ± 905.30	3074.53 ± 1290.81	16.35 ± 11.69
	Non-Hispanic Black	1964 (21.0%)	3591.03 ± 964.72	2884.56 ± 807.72	2963.94 ± 1250.62	17.31 ± 15.51
	Other Races	814 (8.7%)	3842.40 ± 944.11	3096.58 ± 793.89	3125.35 ± 1195.47	18.94 ± 16.09
*P* value			< .001	< .001	< .001	< .001
Education level						
	High school and below	4330 (46.5%)	4030.13 ± 1071.08	3164.72 ± 889.99	3038.29 ± 1313.64	16.10 ± 12.68
	Above high school	4998 (53.5%)	4264.10 ± 1083.92	3357.44 ± 877.82	3185.46 ± 1248.81	17.08 ± 12.69
*P* value			< .001	< .001	< .001	< .001
Marital status						
	Married	5088 (54.5%)	4170.82 ± 1062.38	3238.28 ± 846.70	2990.59 ± 1209.43	16.69 ± 11.00
	Others	4240 (45.5%)	4187.64 ± 1116.66	3354.93 ± 937.84	3331.99 ± 1338.08	16.77 ± 14.78
*P* value			.461	< .001	< .001	.771
PIR						
	< 1.3	2421 (25.9%)	4038.82 ± 1067.62	3234.19 ± 890.29	3254.62 ± 1307.20	16.33 ± 15.04
	1.3–3.5	3160 (33.8%)	4108.67 ± 1075.60	3250.82 ± 900.47	3148.10 ± 1328.05	16.60 ± 11.87
	> 3.5	3747 (40.1%)	4267.48 ± 1088.74	3326.01 ± 875.86	3079.41 ± 1226.17	16.93 ± 12.34
*P* value			< .001	< .001	< .001	.223
BMI(kg/m^2^)						
	< 25	2702 (28.9%)	4189.56 ± 1032.53	3315.27 ± 869.67	3145.65 ± 1302.45	16.20 ± 13.40
	25–30	3224 (34.5%)	4337.02 ± 1098.39	3381.66 ± 892.57	3148.65 ± 1267.98	17.06 ± 12.63
	>30	3402 (36.4%)	4001.71 ± 1091.31	3161.03 ± 882.83	3099.80 ± 1256.64	16.84 ± 12.07
*P* value			< .001	< .001	.234	.024
Smoking						
	Yes	3854 (41.3%)	4251.16 ± 1082.56	3269.72 ± 906.06	2942.50 ± 1343.31	15.49 ± 10.94
	No	5474 (58.6%)	4127.60 ± 1083.96	3297.63 ± 873.94	3259.98 ± 1209.55	17.56 ± 13.70
*P* value			< .001	.1357	< .001	< .001
Occupational exposure						
	Yes	4771 (51.1%)	4455.73 ± 1070.71	3486.69 ± 886.90	3275.75 ± 1317.28	16.64 ± 12.29
	No	4557 (48.8%)	3888.94 ± 1022.70	3078.13 ± 838.26	2980.90 ± 1211.60	16.80 ± 13.10
*P* value			< .001	< .001	< .001	.527
Excessive drinking						
	Yes	1167 (12.5%)	3650.88 ± 1000.14	2906.62 ± 829.08	2916.82 ± 1203.16	16.60 ± 10.71
	No	8161 (87.5%)	4239.44 ± 1077.89	3330.77 ± 883.17	3156.24 ± 1280.92	16.73 ± 12.91
*P* value			< .001	< .001	< .001	.763
Setting time (per day)						
	< 4h	4468 (47.9%)	4170.53 ± 1082.66	3278.94 ± 889.37	3127.17 ± 1296.33	16.61 ± 12.63
	4–6h	1934 (20.7%)	4093.14 ± 1065.62	3214.87 ± 881.04	3044.98 ± 1279.49	16.29 ± 11.79
	6–8h	1365 (14.6%)	4170.06 ± 1073.83	3297.92 ± 867.55	3179.00 ± 1245.13	16.16 ± 10.61
	> 8h	1561 (16.8%)	4285.00 ± 1109.47	3365.37 ± 897.62	3190.14 ± 1244.71	17.82 ± 14.95
*P* value			< .001	< .001	.002	< .001
Diabetes						
	Yes	1089 (11.6%)	3635.67 ± 1033.86	2809.25 ± 817.64	2628.52 ± 1185.67	17.57 ± 11.86
	No	8239 (88.4%)	4227.67 ± 1076.04	3330.25 ± 880.45	3177.44 ± 1273.08	16.64 ± 12.76
*P* value			< .001	< .001	< .001	.049
Hypertension						
	Yes	2800 (20.0%)	3872.71 ± 1103.40	2961.58 ± 879.05	2655.86 ± 1241.89	17.77 ± 12.90
	No	6528 (80.0%)	4287.28 ± 1057.17	3402.92 ± 860.74	3301.82 ± 1243.24	16.34 ± 12.60
*P* value			< .001	< .001	< .001	< .001
Total energy intake (kcal/day)						
	Q1 229–1513.5	2331 (25.0%)	3639.50 ± 967.98	2888.15 ± 816.26	2858.12 ± 1214.20	15.73 ± 11.99
	Q2 1514–1962.5	2333 (25.0%)	3925.47 ± 991.94	3079.41 ± 812.09	2912.67 ± 1208.33	16.42 ± 11.64
	Q3 1963–2532.5	2332 (25.0%)	4237.03 ± 1040.47	3330.97 ± 859.65	3155.73 ± 1286.16	17.14 ± 14.14
	Q4 2533–13,509	2332 (25.0%)	4770.16 ± 997.67	3742.35 ± 819.93	3519.93 ± 1270.82	17.36 ± 12.62
*P* value			< .001	< .001	< .001	< .001

BMI = body mass index, FEF25–75% = forced expiratory flow between 25% and 75% of vital capacity, FeNO = fractional exhaled nitric oxide, FEV1 = forced expiratory volume in the first second, FVC = forced vital capacity, N = number of participants, PIR = family income–poverty ratio, SD = standard deviation.

Table 2 displays the outcomes stratified by quartiles of fat-soluble vitamin intake. As the intake of vitamins A, D, E, and K increased, FVC and FEV1 generally improved. FeNO increased with higher intake of vitamins A, E, and K.

**Table 2:** Weighted basic characteristics of FeNO and lung function parameters by fat-soluble vitamin.

Characteristic		Total	FVC (mL)	FEV1 (mL)	FEF25%75% (mL/s)	FeNO (ppb)
		N = 9328	Mean ± SD			
Vitamin A (µg/day)						
	Q1 (0–315)		4038.99 ± 1054.91	3219.01 ± 885.33	3192.98 ± 1288.56	15.53 ± 12.65
	Q2 (316–516)		4083.01 ± 1066.63	3224.37 ± 874.18	3114.46 ± 1273.32	16.25 ± 12.00
	Q3 (517–788)		4157.02 ± 1062.14	3258.35 ± 865.88	3068.84 ± 1253.08	16.80 ± 11.97
	Q4 (789–16,122)		4372.95 ± 1113.56	3410.02 ± 905.08	3156.00 ± 1283.47	17.87 ± 13.76
*P* value			<0.001	<0.001	0.008	<0.001
**Vitamin D** **(µg/day**)						
	Q1 (0–1.8)		4078.14 ± 1050.20	3237.88 ± 881.06	3176.16 ± 1283.87	16.27 ± 13.94
	Q2 (1.9–3.5)		4093.48 ± 1082.75	3208.69 ± 867.55	3038.29 ± 1225.35	16.68 ± 12.80
	Q3 (3.6–6.1)		4119.20 ± 1078.65	3236.96 ± 886.27	3068.25 ± 1272.35	16.84 ± 11.89
	Q4 (6.2–70.6)		4406.89 ± 1092.37	3452.58 ± 891.03	3237.64 ± 1305.78	17.07 ± 12.07
*P* value			< .001	< .001	< .001	.176
Vitamin E (mg/day)						
	Q1 (0–4.5)		3882.53 ± 1052.17	3084.07 ± 882.94	3062.71 ± 1279.59	15.95 ± 12.95
	Q2 (4.6–6.6)		4075.57 ± 1052.91	3223.27 ± 866.87	3109.45 ± 1249.86	16.40 ± 11.98
	Q3 (6.7–9.6)		4203.76 ± 1057.92	3300.46 ± 861.23	3123.97 ± 1282.70	16.91 ± 14.02
	Q4 (9.7–81.8)		4439.62 ± 1092.97	3463.34 ± 893.34	3201.85 ± 1281.96	17.34 ± 11.77
*P* value			< .001	< .001	.002	.001
Vitamin K (µg/day)						
	Q1 (0–41.2)		4021.71 ± 1042.65	3186.67 ± 862.68	3122.43 ± 1250.67	15.83 ± 12.23
	Q2 (41.3–68.6)		4189.50 ± 1094.07	3312.77 ± 891.62	3201.70 ± 1268.79	16.79 ± 14.88
	Q3 (68.7–121.4)		4259.24 ± 1093.84	3338.12 ± 894.59	3146.77 ± 1321.27	16.58 ± 11.78
	Q4 (121.5–1880.9)		4210.02 ± 1088.55	3290.06 ± 888.74	3059.63 ± 1251.09	17.47 ± 11.64
*P* value			< .001	< .001	.001	< .001

FEF25–75% = forced expiratory flow between 25% and 75% of vital capacity, FeNO = fractional exhaled nitric oxide, FEV1 = forced expiratory volume in the first second, FVC = forced vital capacity, N = number of participants, SD = standard deviation.

Collinearity screening (Table 3) showed all VIFs were < 5, indicating no significant collinearity.

**Table 3:** Screening for collinearity among covariates.

Covariates	Variance Inflation Factor
Vitamins A	1.5
Vitamins D	1.3
Vitamins E	1.6
Vitamins K	1.3
Age	1.3
Gender	1.4
Race	1.1
Education level	1.2
Marital status	1.1
PIR	1.2
BMI (kg/m^2^)	1.1
Smoking	1.1
Occupational exposure	1.2
Excessive drinking	1.0
Setting time (per day)	1.1
Diabetes	1.1
Hypertension	1.3
Total energy intake (kcal/day)	1.8

BMI = body mass index, PIR = family income–poverty ratio.

### 3.2. Relationship and sensitivity analysis between fat-soluble vitamins and lung function

Table 4 presents the associations with FVC. In the fully adjusted model (Model 3), a significant positive association was observed for vitamin A (*β* = 0.04 mL per µg/day, 95% confidence interval [CI]: 0.01–0.06; *P* = .021) and vitamin D (*β* = 3.72 mL per µg/day, 95% CI: 0.66–6.78; *P* = .017). While continuous vitamin E and K were not significant in Model 3, the quartile trend tests were significant (*P* for trend = .042 and .021, respectively), suggesting positive correlations.

**Table 4:** Association between vitamins A, D, E, K, and FVC.

Exposure		Model1 *β* (95% CI)	Model 2 *β* (95% CI)	Model 3 *β* (95% CI)
		*P*	*P*	*P*
Vitamin A (µg/day)		0.21 (0.16–0.25)	0.09 (0.07–0.12)	0.04 (0.01–0.06)
		< .001	< .001	.0202
Group				
	Quartile 1			
	Quartile 2	44.03 (−21.37–109.43)	59.03 (17.65–100.41)	21.44 (−19.56–62.45)
	*P*	.187	.005	.305
	Quartile 3	118.04 (53.59–182.48)	90.91 (50.03–131.80)	25.35 (−16.02–66.71)
	*P*	< .001	< .001	.229
	Quartile 4	333.97 (271.23–396.70)	175.47 (135.23–215.71)	73.96 (31.01–116.90)
	*P*	< .001	< .001	< .001
*P* for trend		0.41 (0.35–0.48) < 0.001	0.20 (0.16–0.25) < 0.001	0.09 (0.04–0.13) 0.0004
*β* (95% CI) for trend		−0.02 (−0.10–0.07)	0.04 (−0.03–0.10)	−0.01 (−0.08–0.06)
Vitamin D (µg/day)		28.15 (23.46–32.84)	8.57 (5.59–11.56)	3.72 (0.66–6.78)
		< .001	< .001	.017
Group				
	Quartile 1			
	Quartile 2	15.33 (−47.00–77.67)	36.61 (−2.72–75.95)	20.07 (−18.63–58.76)
	*P*	.629	.068	.309
	Quartile 3	41.05 (−21.31–103.41)	24.79 (−14.66–64.25)	−6.75 (−45.89–32.39)
	*P*	.197	.218	.735
	Quartile 4	328.74 (267.12–390.37)	122.48 (83.30–161.65)	58.70 (18.67–98.73)
	*P*	< .001	< .001	.004
*P* for trend		< .001	< .001	.006
*β* (95% CI) for trend		44.80 (37.15–52.45)	15.25 (10.38–20.11)	6.99 (2.01–11.96)
Vitamin E (mg/day)		33.50 (29.38–37.62)	9.48 (6.82–12.13)	1.49 (−1.55–4.53)
		< .001	< .001	.338
Group				
	Quartile 1			
	Quartile 2	193.04 (127.77–258.32)	66.53 (24.96–108.10)	11.69 (−30.01–53.39)
	*P*	< .001	.002	.582
	Quartile 3	321.23 (257.27–385.19)	105.33 (64.39–146.27)	16.72 (−26.33–59.77)
	*P*	< .001	< .001	.4466
	Quartile 4	557.09 (494.87–619.31)	170.80 (130.36–211.25)	45.50 (−1.36–92.36)
	*P*	< .001	< .001	.057
*P* for trend		< .001	< .001	.042
*β* (95% CI) for trend		59.07 (52.69–65.44)	17.78 (13.63–21.93)	5.02 (0.18–9.86)
Vitamin K (µg/day)		0.05 (−0.11–0.22)	0.25 (0.15–0.35)	0.08 (−0.02–0.19)
		.534	< .001	.117
Group				
	Quartile 1			
	Quartile 2	167.79 (102.98–232.61)	73.78 (33.06–114.50)	21.27 (−19.41–61.96)
	*P*	< .001	< .001	.305
	Quartile 3	237.54 (173.31–301.76)	108.15 (67.68–148.62)	32.96 (−8.61–74.52)
	*P*	< .001	< .001	.120
	Quartile 4	188.31 (124.99–251.63)	151.18 (111.02–191.35)	52.13 (9.84–94.43)
	*P*	< .001	< .001	.016
*P* for trend		< .001	< .001	.021
*β* (95% CI) for trend		0.73 (0.37–1.09)	0.79 (0.56–1.01)	0.28 (0.04–0.51)

CI = confidence interval, FVC = forced vital capacity.

Table 5 shows the associations with FEV1. In the fully adjusted Model 3, continuous vitamins were not significantly associated with FEV1. However, quartile sensitivity analyses revealed significant positive trends for vitamins A, D, and K (*P* for trend = .012, .011, and .008, respectively).

**Table 5:** Association between Vitamins A, D, E, K, and FEV1.

Exposure		Model1 *β* (95% CI)	Model 2 *β* (95% CI)	Model 3 *β* (95% CI)
		*P*	*P*	*P*
Vitamin A (µg/day)		0.12 (0.09–0.16)	0.06 (0.04–0.09)	0.02 (−0.00–0.05)
		< .001	< .001	.081
Group				
	Quartile 1			
	Quartile 2	5.36 (−48.29–59.01)	36.40 (2.69–70.11)	8.67 (−24.89–42.23)
	*P*	.845	.034	.612
	Quartile 3	39.34 (−13.53–92.20)	53.27 (19.97–86.57)	5.19 (−28.67–39.04)
	*P*	.145	.002	.764
	Quartile 4	191.01 (139.55–242.47)	116.85 (84.07,149.63)	41.60 (6.45–76.75)
	*P*	< .001	< .001	.021
*P* for trend		< .001	< .001	.012
*β* (95% CI) for trend		0.25 (0.19–0.30)	0.14 (0.10–0.17)	0.05 (0.01–0.09)
Vitamin D (µg/day)		18.21 (14.36–22.05)	5.03 (2.60–7.47)	1.72 (−0.79–4.22)
		< .001	< .001	.179
Group				
	Quartile 1			
	Quartile 2	−29.20 (−80.25–21.86)	2.13 (−29.89–34.14)	−9.32 (−40.98–22.34)
	*P*	.262	.896	.564
	Quartile 3	−0.93 (−52.00–50.15)	7.03 (−25.08–39.14)	−15.75 (−47.77–16.27)
	*P*	.971	.668	.335
	Quartile 4	214.70 (164.23–265.17)	80.03 (48.15–111.91)	35.75 (3.00–68.50)
	*P*	< .001	< .001	.032
*P* for trend		< .001	< .001	.011
*β* (95% CI) for trend		30.92 (24.65–37.19)	10.95 (6.99–14.91)	5.26 (1.19–9.33)
Vitamin E (mg/day)		22.76 (19.38–26.14)	5.94 (3.78–8.10)	0.79 (−1.70–3.28)
		< .001	< .001	.534
Group				
	Quartile 1			
	Quartile 2	139.20 (85.52–192.87)	49.30 (15.44–83.16)	13.29 (−20.82–47.41)
	*P*	< .001	.004	.445
	Quartile 3	216.39 (163.80–268.99)	69.90 (36.56–103.25)	12.92 (−22.30–48.14)
	*P*	< .001	< .001	.472
	Quartile 4	379.27 (328.11–430.43)	115.94 (82.99–148.88)	36.85 (−1.49–75.18)
	*P*	< .001	< .001	.059
*P* for trend		< .001	< .001	.054
*β* (95% CI) for trend		39.86 (34.62–45.10)	11.88 (8.50–15.26)	3.88 (−0.08–7.83)
Vitamin K (µg/day)		−0.05 (−0.18–0.09)	0.18 (0.10–0.27)	0.07 (−0.02–0.15)
		.472	< .001	.114
Group				
	Quartile 1			
	Quartile 2	126.10 (73.04–179.16)	70.24 (37.10–103.37)	38.26 (4.98–71.54)
	*P*	< .001	< .001	.024
	Quartile 3	151.45 (98.88–204.02)	80.41 (47.48–113.35)	36.30 (2.31–70.30)
	*P*	< .001	< .001	.036
	Quartile 4	103.39 (51.56–155.22)	119.60 (86.92–152.28)	56.95 (22.35–91.54)
	*P*	< .001	< .001	.001
*P* for trend		.044	< .001	.008
*β* (95% CI) for trend		0.30 (0.01–0.59)	0.59 (0.41–0.78)	0.26 (0.07–0.45)

CI = confidence interval, FEV1 = forced expiratory volume in the first second.

As shown in Table 6, no statistically significant associations were found between any fat-soluble vitamin and FEF25–75% in the fully adjusted models (p for trend > 0.05).

**Table 6:** Association between Vitamins A, D, E, K, and FEF25%–75%.

Exposure		Model1 *β* (95% CI)	Model 2 *β* (95% CI)	Model 3 *β* (95% CI)
		*P*	*P*	*P*
Vitamin A (µg/day)		−0.01 (−0.06–0.04)	0.02 (−0.03–0.06)	−0.01 (−0.05–0.04)
		.648	.458	.788
Group				
	Quartile 1			
	Quartile 2	−78.52 (−155.90–−1.14)	7.76 (−52.67–68.20)	−7.14 (−67.68–53.39)
	*P*	.046	.801	.817
	Quartile 3	−124.14 (−200.39–−47.89)	−13.38 (−73.09–46.32)	−38.03 (−99.10–23.03)
	*P*	< .001	.660	.222
	Quartile 4	−36.98 (−111.21–37.24)	34.14 (−24.62–92.91)	−7.66 (−71.06–55.74)
	*P*	.328	.254	.812
*P* for trend		.720	.232	.846
*β* (95% CI) for trend		−0.02 (−0.10–0.07)	0.04 (−0.03–0.10)	−0.01 (−0.08–0.06)
Vitamin D (µg/day)		5.27 (−0.28–10.83)	−1.07 (−5.42–3.28)	−2.18 (−6.70–2.33)
		.062	.630	.343
Group				
	Quartile 1			
	Quartile 2	−137.88 (−211.57–−64.19)	−73.98 (−131.29–−16.67)	−80.75 (−137.85–−23.66)
	*P*	< .001	.011	.005
	Quartile 3	−107.91 (−181.63–−34.19)	−48.67 (−106.15–8.81)	−63.65 (−121.40–−5.90)
	*P*	.004	.097	.030
	Quartile 4	61.48 (−11.37–134.32)	7.90 (−49.18–64.97)	−10.65 (−69.72–48.41)
	*P*	.098	.786	.723
*P* for trend		.002	.239	.576
*β* (95% CI) for trend		14.17 (5.12–23.22)	4.26 (−2.83–11.35)	2.09 (−5.25–9.43)
Vitamin E (mg/day)		8.73 (3.83–13.63)	−1.08 (−4.96–2.79)	−2.30 (−6.79–2.19)
		< .001	.583	.315
Group				
	Quartile 1			
	Quartile 2	46.74 (−31.27–124.75)	−7.89 (−68.59–52.81)	−19.72 (−81.25–41.81)
	*P*	.240	.798	.530
	Quartile 3	61.26 (−15.18–137.70)	−9.96 (−69.74–49.82)	−24.11 (−87.63–39.42)
	*P*	.116	.744	.457
	Quartile 4	139.14 (64.78–213.49)	4.67 (−54.38–63.72)	−11.29 (−80.43–57.85)
	*P*	< .001	.876	.749
*P* for trend		< .001	.771	.926
*β* (95% CI) for trend		14.77 (7.15–22.38)	0.90 (−5.16–6.96)	−0.34 (−7.47–6.80)
Vitamin K (µg/day)		−0.38 (−0.57–−0.19)	0.02 (−0.13–0.18)	−0.00 (−0.16–0.15)
		< .001	.746	.955
Group				
	Quartile 1			
	Quartile 2	79.27 (2.93–155.61)	76.91 (17.53–136.29)	75.12 (15.09–135.14)
	*P*	.041	.011	.014
	Quartile 3	24.34 (−51.30–99.98)	38.17 (−20.85–97.19)	42.68 (−18.64–104.00)
	*P*	.528	.205	.172
	Quartile 4	−62.80 (−137.37–11.77)	69.87 (11.30–128.44)	62.32 (−0.08–124.72)
	*P*	.0989	.0194	.0503
*P* for trend		.002	.133	.297
*β* (95% CI) for trend		−0.66 (−1.08–−0.24)	0.25 (−0.08–0.58)	0.18 (−0.16–0.53)

CI = confidence interval, FEF25–75% = forced expiratory flow between 25% and 75% of vital capacity.

### 3.3. Relationship and sensitivity analysis between fat-soluble vitamins and FeNO

A significant positive association was found between vitamin A and K intake and FeNO in all 3 models (Table 7). In the fully adjusted model, each unit increase in vitamin A and K intake was associated with increases of 1.1 ppb (95% CI: 0.54–1.65; *P* < .001) and 3.3 ppb (95% CI: 1.39–5.28; *P* < .001) in FeNO, respectively. Vitamins D and E were not significantly associated with FeNO in the fully adjusted model.

**Table 7:** Association between Vitamins A, D, E, K, and FeNo.

Exposure		*Model1* *β* (95% CI)	Model 2 *β* (95% CI)	Model 3 *β* (95% CI)
		*P*	*P*	*P*
Vitamin A (mg/day)		1.41 (0.89, 1.93)	1.28 (0.76, 1.80)	1.10 (0.54–1.65)
		< .001	< .001	< .001
Group				
	Quartile 1			
	Quartile 2	0.72 (−0.05–1.49)	0.91 (0.15–1.68)	0.76 (−0.01–1.53)
	*P*	.066	.019	.052
	Quartile 3	1.27 (0.51–2.03)	1.29 (0.54–2.05)	1.18 (0.40–1.96)
	*P*	.001	< .001	.003
	Quartile 4	2.34 (1.60–3.08)	2.33 (1.59–3.07)	2.10 (1.29–2.90)
	*P*	< .001	< .001	< .001
*P* for trend		< .001	< .001	< .001
*β* (95% CI) for trend		2.72 (1.90–3.54)	2.62 (1.80–3.44)	2.37 (1.48–3.27)
Vitamin D (mg/day)		99.41 (44.17–154.65)	61.84 (6.77–116.90)	43.68 (−13.74–101.10)
		< .001	.028	.136
Group				
	Quartile 1			
	Quartile 2	0.41 (−0.32–1.15)	0.30 (−0.42–1.03) 0.4110	0.18 (−0.55–0.90)
	*P*	.271	.068	.635
	Quartile 3	0.57 (−0.17–1.31)	0.40 (−0.33–1.13) 0.2824	0.21 (−0.53–0.94)
	*P*	.1287	.2181	.5840
	Quartile 4	0.80 (0.07–1.53)	0.34 (−0.38–1.07)	0.05 (−0.71–0.80)
	*P*	.030	.350	.904
*P* for trend		.037	.426	.987
*β* (95% CI) for trend		95.52 (5.40–185.63)	36.39 (−53.35–126.13)	−0.73 (−94.05–92.59)
Vitamin E (g/day)		70.29 (21.49–119.08)	45.49 (−3.52–94.50)	25.36 (−31.71–82.44)
		.004	.068	.383
Group				
	Quartile 1			
	Quartile 2	0.45 (−0.33–1.23)	0.40 (−0.37–1.17)	0.26 (−0.52–1.04)
	*P*	.2561	.3067	.5180
	Quartile 3	0.96 (0.20–1.72)	0.70 (−0.06–1.45)	0.55 (−0.26–1.36)
	*P*	.0135	.0716	.1823
	Quartile 4	1.39 (0.65–2.13)	0.98 (0.23–1.73)	0.77 (−0.11–1.65)
	*P*	< .001	.010	.084
*P* for trend		.001	.009	.078
*β* (95% CI) for trend		149.53 (73.74–225.33)	101.37 (24.70–178.04)	81.37 (−9.36–172.10)
Vitamin K (mg/day)		4.30 (2.38–6.22)	3.97 (2.06–5.87)	3.33 (1.39–5.28)
		< .001	< .001	< .001
Group				
	Quartile 1			
	Quartile 2	0.95 (0.19–1.71)	0.78 (0.03–1.53)	0.71 (−0.05–1.48)
	*P*	.0141	.0427	.0667
	Quartile 3	0.74 (−0.01–1.49)	0.41 (−0.34–1.15)	0.30 (−0.48–1.08)
	*P*	.0537	.2847	.4464
	Quartile 4	1.64 (0.89–2.38)	1.32 (0.58–2.06)	1.02 (0.23–1.81)
	*P*	< .001	< .001	.012
*P* for trend		< .001	.002	.031
*β* (95% CI) for trend		8.31 (4.13–12.50)	6.77 (2.59–10.95)	4.84 (0.44–9.24)

CI = confidence interval, FeNO = fractional exhaled nitric oxide.

### 3.4. Nonlinear relationships between fat-soluble vitamins and lung function

**Figure 2.**
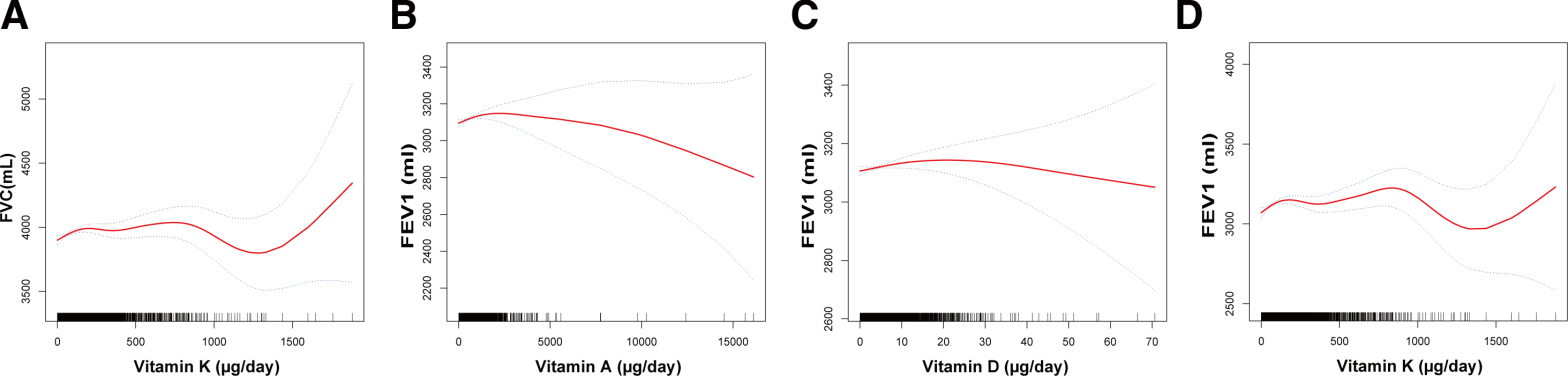
Smooth curve fitting: the nonlinear relationships among vitamin A, vitamin D, vitamin K and FVC/FEV1. FEV1 = forced expiratory volume in the first second, FVC = forced vital capacity.

Exploratory analyses were conducted to investigate potential nonlinear relationships, given the lack of statistical significance in fully adjusted linear models but significant trends in quartile-based sensitivity analyses for some vitamin-outcome pairs. GAMs demonstrated inverted U-shaped nonlinear relationships for vitamins A and D with FEV1 (Fig. 2). The threshold levels were 958 µg/day for vitamin A and 11.95 µg/day for vitamin D (Table 8). Below these thresholds, each unit increase in vitamin A and vitamin D intake was associated with an increase in FEV1 of 0.07 mL (95% CI: 0.02–0.11) and 4.15 mL (95% CI: 0.39–7.91), respectively. No significant nonlinear relationship was observed between vitamin K and FVC. These nonlinear and threshold results are exploratory and require validation in independent cohorts.

**Table 8:** Threshold effect analysis between Vitamins A, D, and FEV1 using the maximum likelihood method, with Bootstrap resampling method to determine CI.

Exposure	Model 3	
	*β* (95% CI)	*P* value
Vitamin A		
< 958 µg/day	0.07 (0.02,0.11)	.004
> 958 µg/day	−0.02 (−0.04,0,01)	.187
Vitamin D		
< 11.95 µg/day	4.15 (0.39,7.91)	.031
> 11.95 µg/day	−2.80 (−8.23,2.64)	.313

CI = confidence interval, FEV1 = forced expiratory volume in the first second.

### 3.5. Subgroup analysis

**Figure 3.**
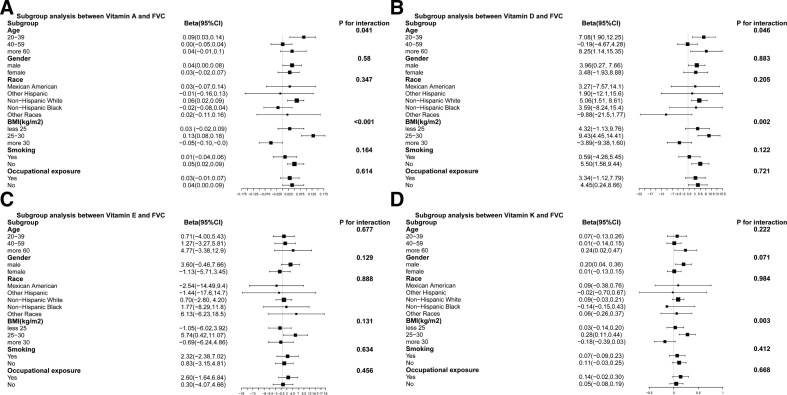
Subgroup analysis of vitamins A, D, E, and K and the FVC. BMI = body mass index, CI = confidence interval, FVC = forced vital capacity.

**Figure 4.**
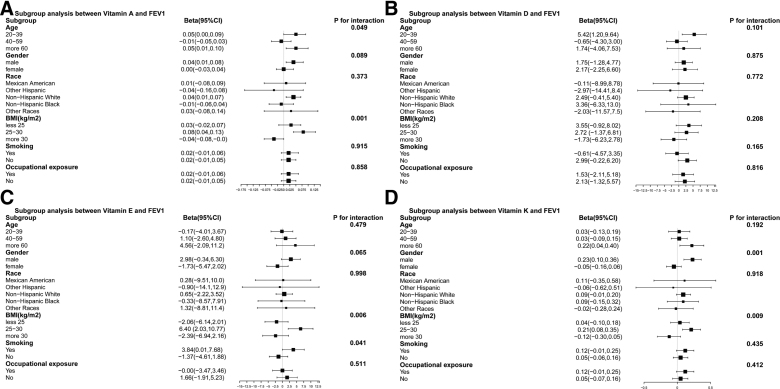
Subgroup analysis of the associations between vitamins A, D, E, and K and FEV1. BMI = body mass index, CI = confidence interval, FEV1 = forced expiratory volume in the first second.

**Figure 5.**
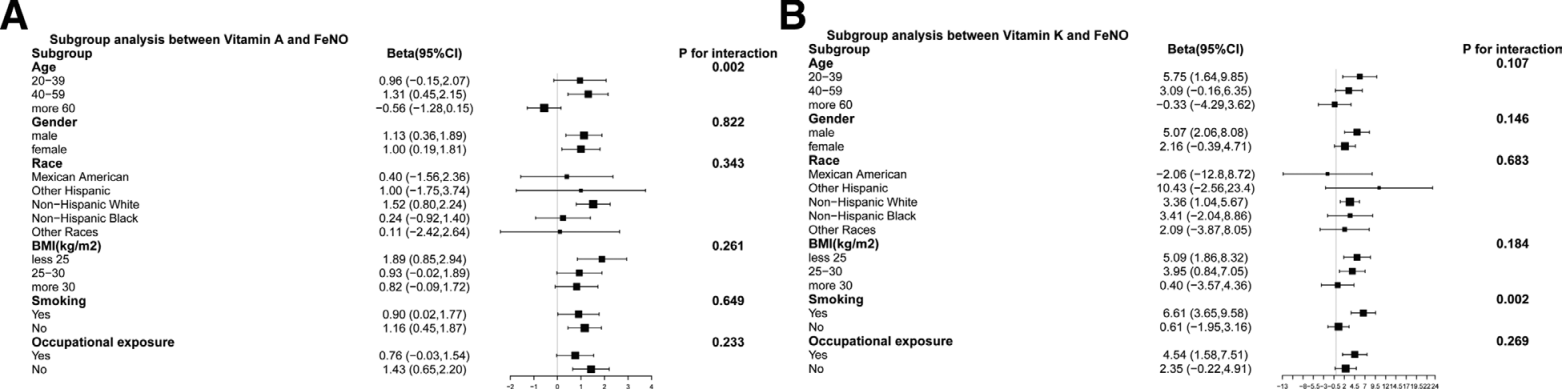
Subgroup analyses of vitamin A, K and FeNO. BMI = body mass index, CI = confidence interval, FeNO = fractional exhaled nitric oxide.

Subgroup analyses and interaction tests for associations with FVC and FEV1 are shown in Figures 3 and 4. BMI-stratified analysis revealed significant interactions for vitamins A, D, and K with FVC (*P* for interaction < .05), and for vitamins A, E, and K with FEV1 (*P* for interaction < .05), particularly among overweight individuals (BMI 25–30 kg/m^2^). For FeNO (Fig. 5), a significant interaction was found between vitamin A and age (*P* for interaction < .05), and between vitamin K and smoking status (*P* for interaction < .05), with the association being more pronounced among smokers.

## 4. Discussion

This study aimed to explore the associations between fat-soluble vitamin intake and lung function, as well as airway inflammation, in a representative sample of U.S. adults. Our cross-sectional analysis found that higher intakes of vitamins A and D were positively associated with lung function (FVC and FEV1), which aligns with observational patterns reported in previous population studies.^[10,11]^ Interestingly, exploratory analyses identified inverted U-shaped relationships for vitamins A and D with FEV1. In contrast, vitamins A and K were positively associated with FeNO, a marker of airway inflammation, presenting a complex pattern that warrants further exploration. Subgroup analyses revealed that BMI significantly modified the associations between several vitamins and lung function, particularly among overweight individuals.

The positive associations between vitamins A and D and pulmonary function observed in our study are consistent with a growing body of literature. Vitamin D has been linked to better FEV1 and FVC in both general populations and individuals with respiratory diseases.^[11]^ Similarly, vitamin A, crucial for maintaining epithelial integrity, has been associated with better lung function.^[4,12]^ The observed positive association between vitamin D and lung function may be related to its biological roles in immune modulation and the mitigation of oxidative stress, such as through the inhibition of the nuclear factor kappa-light-chain-enhancer of activated B cells signaling pathway.^[13,14]^ Vitamin A, via its active form retinoic acid, regulates cellular differentiation and immune responses, contributing to epithelial homeostasis and potentially mitigating fibrotic changes.^[15,16]^

The positive association between vitamins A and K and FeNO was an unexpected finding. The underlying mechanisms remain unclear and are speculative. It could be related to the pro-inflammatory effects of very high-dose vitamin A, though our data do not confirm this. Alternatively, it might be due to residual confounding by other components in foods rich in these vitamins.^[17]^ These mechanisms are hypothetical and cannot be validated in this cross-sectional analysis; future experimental or prospective studies are needed to explore these causal pathways.

The inverted U-shaped relationships between vitamins A/D and FEV1, along with the identified threshold values (958 µg/day for vitamin A and 11.95 µg/day for vitamin D), are exploratory findings from our analysis. These thresholds are sensitive to model specification and covariate adjustment, and their clinical relevance is uncertain. They should be interpreted as hypothesis-generating rather than definitive dietary guidance. Vitamin K’s role in lung health may be mediated through its activation of matrix gla protein, which inhibits soft tissue calcification and helps maintain lung elasticity, offering a potential mechanism for the associations observed in our quartile-based analyses.^[18–20]^

Subgroup analyses revealed significant interactions with BMI, particularly among overweight individuals. This suggests that adiposity may modify the relationship between these nutrients and respiratory health, possibly through shared inflammatory pathways.^[15]^ However, given the multiple strata and interaction tests conducted, these findings may be subject to false-positive results and should be validated in independent studies.

The strengths of this study include its large, representative sample size, comprehensive measures of pulmonary function and dietary intake, and adjustment for a wide range of confounders. However, several limitations must be acknowledged. First, dietary intake was assessed via 2 24-hour recalls, which may not reflect long-term usual intake and could introduce measurement bias. Second, we did not measure serum vitamin concentrations, which may better reflect biological availability. Third, the cross-sectional design prevents the establishment of causality or temporal direction. Fourth, residual confounding from unmeasured factors (e.g., physical activity, other dietary components) cannot be excluded.^[6,21]^ Fifth, the exploratory and subgroup analyses carry a risk of false-positive findings due to multiple comparisons.

## 5. Conclusions

In conclusion, our cross-sectional study identifies observational associations between dietary fat-soluble vitamin intake and respiratory health indicators in a representative U.S. adult population: vitamins A and D are positively associated with lung function (FVC and FEV1), while vitamins A and K are positively associated with FeNO. Exploratory analyses suggest inverted U-shaped relationships between vitamins A/D and FEV1, but these findings are hypothesis-generating and require validation. Our results provide population-level observational evidence for future research on nutrition and respiratory health, but no causal or preventive implications can be drawn from this cross-sectional analysis. Further prospective or interventional studies are needed to clarify the mechanisms and clinical relevance of these associations.

## Acknowledgements

The authors would like to thank all the participants and staff of the National Health and Nutrition Examination Survey (NHANES) for their valuable contributions to this study. We also extend our gratitude to the National Center for Health Statistics (NCHS) for providing the data.

## Author contributions

**Conceptualization:** Hanyan Xu, Qingyuan Zhan.

**Data curation:** Ping Zhang, Shengsong Chen, Hanyan Xu, Yinshui Miao, Qingyuan Zhan.

**Formal analysis:** Ping Zhang.

**Funding acquisition:** Qingyuan Zhan.

**Investigation:** Ping Zhang, Hanyan Xu, Qingyuan Zhan.

**Methodology:** Ping Zhang, Hanyan Xu.

**Resources:** Ping Zhang.

**Software:** Ping Zhang, Shengsong Chen, Yinshui Miao, Hui Yang.

**Supervision:** Ping Zhang, Shengsong Chen, Hanyan Xu, Yinshui Miao, Hui Yang.

**Validation:** Ping Zhang, Shengsong Chen, Hui Yang.

**Visualization:** Ping Zhang, Shengsong Chen, Hanyan Xu, Yinshui Miao, Hui Yang.

**Writing – original draft:** Ping Zhang, Shengsong Chen, Hanyan Xu, Qingyuan Zhan.

**Writing – review & editing:** Ping Zhang, Qingyuan Zhan.
